# Generalizability of Machine Learning Models: Quantitative Evaluation of Three Methodological Pitfalls

**DOI:** 10.1148/ryai.220028

**Published:** 2022-11-16

**Authors:** Farhad Maleki, Katie Ovens, Rajiv Gupta, Caroline Reinhold, Alan Spatz, Reza Forghani

**Affiliations:** From the Department of Computer Science, University of Calgary, Calgary, Canada (F.M., K.O.); Department of Radiology, Massachusetts General Hospital, Boston, Mass (R.G.); Augmented Intelligence & Precision Health Laboratory (AIPHL), Department of Radiology and the Research Institute of the McGill University Health Centre, McGill University, Montreal, Canada (C.R., R.F.); Montreal Imaging Experts, Montreal, Canada (C.R., R.F.); Division of Pathology, Jewish General Hospital, Montreal, Canada (A.S.); and Radiomics and Augmented Intelligence Laboratory (RAIL), Department of Radiology and the Norman Fixel Institute for Neurologic Diseases, University of Florida College of Medicine, UF Health Shands Hospital, 1600 SW Archer Rd, Gainesville, FL 32610-0374 (R.F.).

**Keywords:** Random Forest, Diagnosis, Prognosis, Convolutional Neural Network (CNN), Medical Image Analysis, Generalizability, Machine Learning, Deep Learning, Model Evaluation

## Abstract

**Purpose:**

To investigate the impact of the following three methodological pitfalls on model generalizability: (*a*) violation of the independence assumption, (*b*) model evaluation with an inappropriate performance indicator or baseline for comparison, and (*c*) batch effect.

**Materials and Methods:**

The authors used retrospective CT, histopathologic analysis, and radiography datasets to develop machine learning models with and without the three methodological pitfalls to quantitatively illustrate their effect on model performance and generalizability. F1 score was used to measure performance, and differences in performance between models developed with and without errors were assessed using the Wilcoxon rank sum test when applicable.

**Results:**

Violation of the independence assumption by applying oversampling, feature selection, and data augmentation before splitting data into training, validation, and test sets seemingly improved model F1 scores by 71.2% for predicting local recurrence and 5.0% for predicting 3-year overall survival in head and neck cancer and by 46.0% for distinguishing histopathologic patterns in lung cancer. Randomly distributing data points for a patient across datasets superficially improved the F1 score by 21.8%. High model performance metrics did not indicate high-quality lung segmentation. In the presence of a batch effect, a model built for pneumonia detection had an F1 score of 98.7% but correctly classified only 3.86% of samples from a new dataset of healthy patients.

**Conclusion:**

Machine learning models developed with these methodological pitfalls, which are undetectable during internal evaluation, produce inaccurate predictions; thus, understanding and avoiding these pitfalls is necessary for developing generalizable models.

**Keywords:** Random Forest, Diagnosis, Prognosis, Convolutional Neural Network (CNN), Medical Image Analysis, Generalizability, Machine Learning, Deep Learning, Model Evaluation

*Supplemental material is available for this article.*

Published under a CC BY 4.0 license.

**Summary**

Three major methodological pitfalls in developing machine learning and deep learning models remain undetected during internal evaluation, leading to overoptimistic estimation of model performance and consequent lack of generalizability.

**Key Points**

■ The following methodological pitfalls in model development may prevent generalizability: (*a*) violation of the independence assumption, (*b*) model evaluation with an inappropriate performance indicator or baseline for performance comparison, and (*c*) batch effect.
■ These pitfalls are often undetected using internal model evaluation and may lead to inaccurate predictions and interpretations.
■ Explicit guidelines to avoid these pitfalls are provided.

## Introduction

Medical images are widely used for diagnosis and treatment planning. Manual qualitative evaluation by domain experts is the most common method for analyzing data from these images, which is time-consuming and prone to interobserver and intraobserver variabilities (1,2). Additionally, human interpretation may not fully leverage quantitative features unapparent to the naked eye. Machine learning (ML) and deep learning (DL) have great potential for supplementing and augmenting expert human assessment by acting as a clinical assistant or decision support tool (3–9).

Despite a large body of published work on applications of ML and DL in medicine, very few are clinically deployed, primarily due to lack of model generalizability (10). Factors affecting generalizability include technical variations and lack of standardization in medical practice, differences in patient demographics across centers, patient genotypic and phenotypic characteristics, and tools and methods used for medical data processing and model development (11).

Multiple guidelines aim to ensure the rigor, quality, and reproducibility of ML and DL models when conducting and presenting research (12–16). Whiting et al (15) developed the Quality Assessment of Diagnostic Accuracy Studies (QUADAS) and its extension QUADAS-2 for a systematic review of diagnostic studies. QUADAS-2 assesses risk of bias in patient selection, index test, reference standard, and flow and timing of a diagnostic study to ensure generalizability. Wolff et al (16) designed the Prediction Model Risk of Bias Assessment Tool, or PROBAST, as a series of questions to facilitate systematic review and assessment of potential bias in clinical prediction models. Collins et al (12) developed the Transparent Reporting of a Multivariable Prediction Model for Individual Prognosis or Diagnosis (TRIPOD) guidelines to encourage transparency in reporting prediction models. TRIPOD contains recommendations for expected content and characteristics of the abstract, introduction, methods, results, and discussion sections of scientific ML and DL articles. Mongan et al (14) published the Checklist for Artificial Intelligence in Medical Imaging (CLAIM) to aid authors and reviewers. Like TRIPOD, CLAIM provides high-level recommendations for preparing scientific manuscripts but focuses on medical imaging, and it is one of the most widely used artificial intelligence (AI) checklists in medical imaging.

These guidelines mainly focus on the reporting and reproducibility aspects of research findings and offer minimal to no guidance regarding good methodological practices in medical applications of ML. Few technical guidelines are available (10), and those that are available are often inaccessible to practitioners in the medical domain. Clear guidelines supported by scientific evidence are essential to promote the development of generalizable ML and DL models that may be clinically deployed.

Here, we identify and investigate the following three major categories of methodological errors in ML and DL model development: (*a*) violation of the independence assumption, (*b*) the use of inappropriate performance indicators for model evaluation, and (*c*) the introduction of batch effect. We also provide guidelines for avoiding these pitfalls.

## Materials and Methods

### Datasets

This retrospective, institutional review board–exempt study used several imaging modalities to show that the methodological pitfalls are not specific to one type.

### Head and Neck Squamous Cell Carcinoma CT Dataset

The CT dataset included pretreatment CT scans in 137 patients with head and neck squamous cell carcinoma (HNSCC) who were treated with radiation therapy (17,18). Hereafter, we refer to this dataset as *HNSCC*. The dataset is available from The Cancer Imaging Archive (TCIA) (17–19). Table S1 provides a summary of the clinical endpoints.

### Lung CT Dataset

This dataset includes 120 CT scan series in 60 patients in TCIA, available from the Lung CT Segmentation Challenge 2017 (19–21). We used this dataset to demonstrate methodological pitfalls related to performance metrics for segmentation.

### Digital Histopathologic Analysis Dataset

The histopathologic analysis dataset contains 143 hematoxylin-eosin–stained formalin-fixed paraffin-embedded whole-slide images of lung adenocarcinoma provided by the Department of Pathology and Laboratory Medicine at Dartmouth-Hitchcock Medical Center (22). The dataset includes five histopathologic patterns: solid (51 slides), lepidic (19 slides), acinar (59 slides), micropapillary (nine slides), and papillary (five slides). We used the 110 slides from patients with solid- and acinar-predominant histopathologic patterns, as they were numerous and relatively balanced.

**Figure 1:**
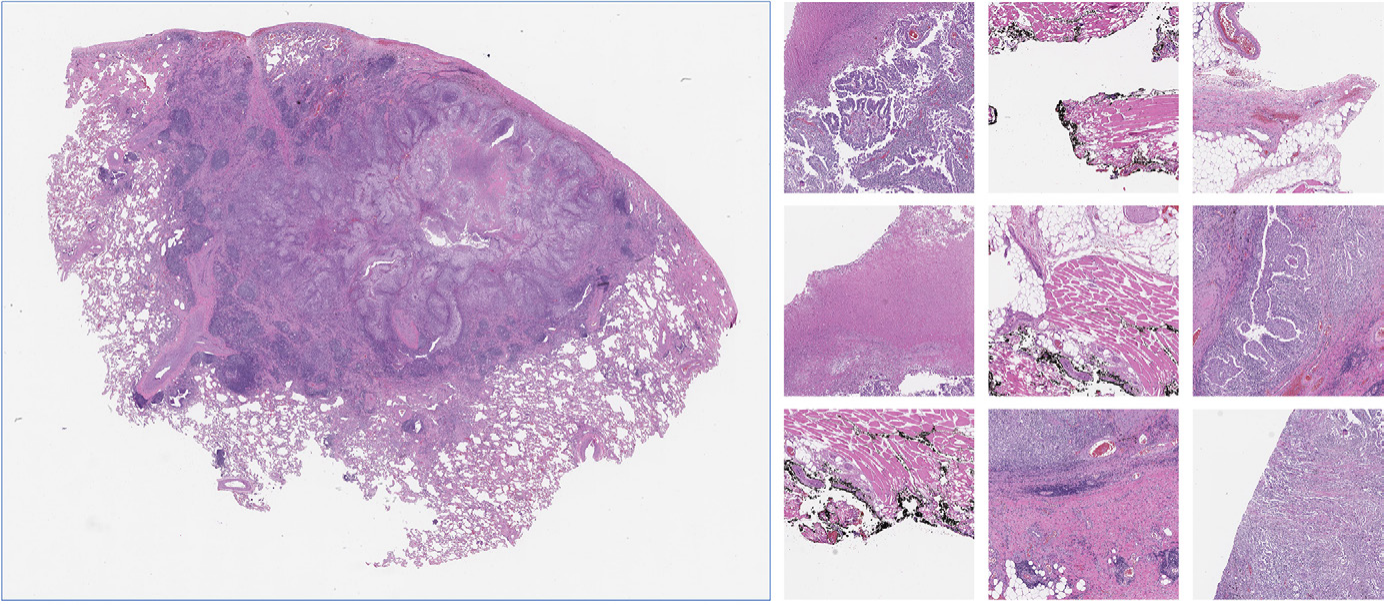
Example of a hematoxylin-eosin–stained whole-slide image, with nine example patches. Image was generated by processing an image extracted from the Dartmouth-Hitchcock Medical Center lung adenocarcinoma dataset, which was scanned with an Aperio AT2 whole-slide scanner at 40× magnification.

Due to the high resolution of the histopathologic images, it was computationally impractical to analyze them as individual whole images (23). Therefore, we first downscaled each image by a factor of 4. Then, using color thresholding, we extracted the foreground, that is, the tissue segments on each slide. Next, for each image, we extracted random patches sized 1024 × 1024 pixels. Patches with 75% or more background were excluded during the patch extraction process. The patch extraction process continued until 200 patches were extracted from each image, resulting in 22 000 patches. Figure 1 illustrates an example whole-slide image, as well as a selection of random patches.

### Chest Radiograph Datasets

**Figure 2:**
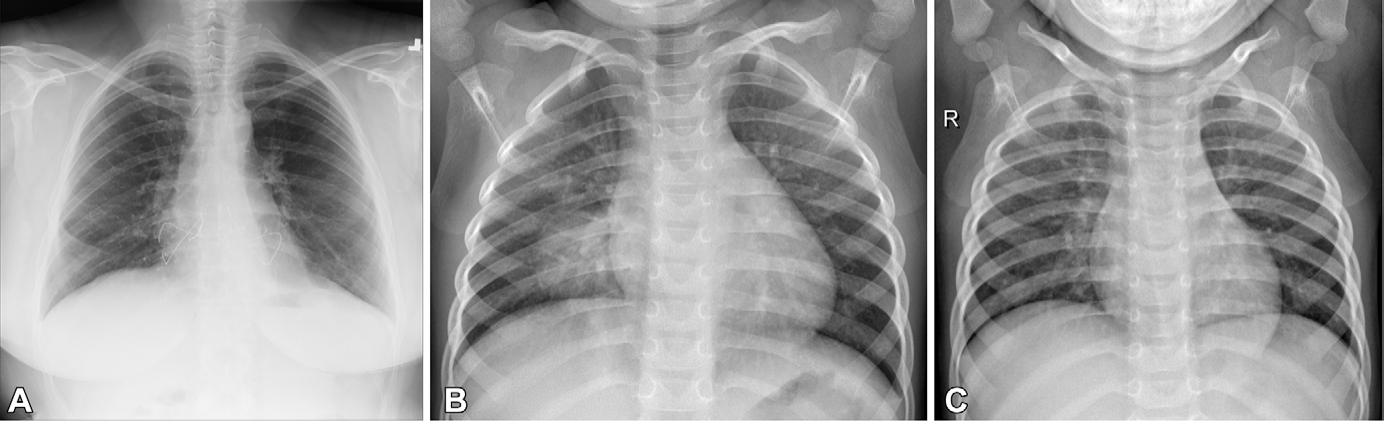
Three example images from the chest radiograph dataset. **(A)** Chest radiograph in a healthy (no findings) adult from the Radiological Society of North America Pneumonia Detection Challenge dataset. **(B)** Chest radiograph of pediatric pneumonia, and **(C)** chest radiograph in a healthy (no findings) pediatric patient.

To demonstrate the impact of batch effects, we used two radiography datasets: 8851 normal chest radiographs with no findings from the Radiological Society of North America (RSNA) Pneumonia Detection Challenge dataset, available on Kaggle (*https://www.kaggle.com/c/rsna-pneumonia-detection-challenge*), and a chest radiograph dataset from Kermany et al (24) that included 1349 normal radiographs with no findings and 3883 radiographs showing pneumonia in pediatric patients. Figure 2 illustrates samples from each dataset.

### Experiments

**Figure 3:**
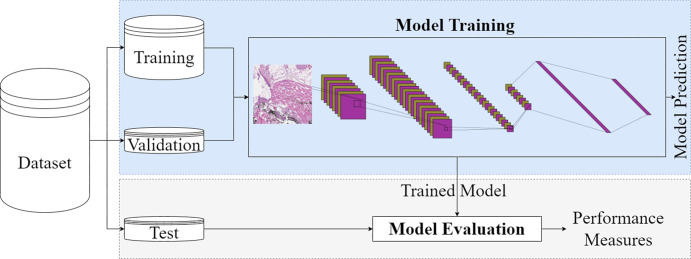
A schematic view of a deep learning pipeline for image analysis.

***Breaking the assumption of independence.—*** ML and DL approaches assume that data used for model training and evaluation are independent and identically distributed. To develop ML models, it is a common practice to split the available data into training, validation, and test sets (Fig 3). The training set is used to learn model parameters, the validation set to select model hyperparameters, and the test set to provide an unbiased estimate of model generalization error. However, the validity of this design is contingent on the assumption of independence.

Data used for model training should be independent of the data used for model evaluation. This assumption can be violated when some data not expected to be available in the prediction and evaluation phase are used for model training, a phenomenon referred to as *data leakage*. We investigated the impact of four common practices associated with data leakage and violation of the independence assumption on model generalizability: (*a*) oversampling, (*b*) data augmentation, (*c*) using several data points from a patient, and (*d*) feature selection.

Oversampling: Class imbalance represents a substantial difference between the number of samples between classes. Often, models developed using imbalanced datasets tend to undermine minority classes. Oversampling can alleviate this challenge by sampling with replacements from the original minority classes to artificially increase the number of samples. Class imbalance is common in medical imaging datasets because of disease rarity and difficulties in imaging certain conditions (25).

To quantitatively show how applying oversampling before splitting data into training, validation, and test sets could affect model generalizability, we evaluated two binary classifiers—as described in Appendix S1—for predicting local recurrence in head and neck cancer by employing a radiomics approach using the HNSCC dataset. The models differed only in the order that oversampling was applied. The first model (model A) was developed by conducting oversampling before data splitting and the second model (model B) by conducting oversampling after data splitting.

Data augmentation:* Data augmentation* refers to computational methods used to generate new data points from existing ones. This technique is commonly used when developing ML and DL models for image analysis, as developing large-scale datasets is often impractical (26), and it can improve the performance and generalizability of resulting models.

To quantitatively show how applying data augmentation before splitting data into training, validation, and test sets could affect model generalizability, we developed two DL-based binary classifiers—as described in Appendix S2—for distinguishing solid- and acinar-predominant histopathologic patterns in patients with lung adenocarcinoma. For these models, every component of the model building and evaluation pipeline was kept the same; however, the first model (model C) was developed by conducting data augmentation before data splitting and the second model (model D) by conducting data augmentation after data splitting.

Using several data points from a patient: To investigate how distributing data points for a patient across training, validation, and test sets could impact model generalizability, we used the pathologic analysis dataset to build two DL-based binary classifiers—as described in Appendix S2—for distinguishing solid- and acinar-predominant histopathologic patterns for patients with lung adenocarcinoma. Model E was developed by randomly distributing the image patches across the training, validation, and test sets. Model F was developed by assigning image patches for each patient to either the training, validation, or test sets. All other model components were identical.

Feature selection: To demonstrate the impact on model generalizability of applying feature selection before splitting data into training, validation, and test sets, we used the HNSCC dataset to develop two binary classifiers—as described in Appendix S1—to predict overall survival using a radiomics approach. The first model (model G) was developed by conducting feature selection before data splitting. The second model (model H) was developed by conducting feature selection after data splitting.

To achieve statistically reliable results for the conventional radiomics analysis, we repeated the model-building process 100 times and calculated the F1 score as the performance measure. We then used the Wilcoxon rank sum test to assess if there was a statistically significant difference between the performance measures derived from model A versus model B, as well as model G versus model H. We used the stats.mannwhitneyu function from the SciPy (version 1.7.3) Python package for statistical analysis, where a *P* value less than .05 was considered as significant.

***Evaluating models with an inappropriate performance indicator or baseline for comparison.—*** Selecting the appropriate quantitative measures for model evaluation is essential for developing predictive models. Setting the baseline for acceptable performance is also required to determine if a model could be used and deployed in real-world settings.

Accuracy, which represents the proportion of samples correctly classified by a model, is commonly used to measure the performance of classification models. We used a model for distant metastasis prediction in the HNSCC dataset to show how accuracy as a performance indicator for imbalanced datasets inappropriately reflects model performance.

We also investigated how selecting an inappropriate baseline for performance comparison impacts interpretations of model outcome. The performance of segmentation models is commonly measured using intersection over union (IoU) and Dice scores. Dice score is a measure of relative overlap and is defined as follows: 
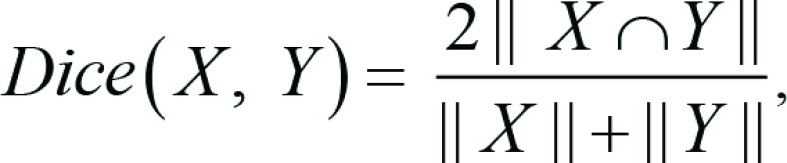
 where *X* and *Y* are two segmentations, for example, the ground truth and the model prediction. IoU is calculated as follows: 
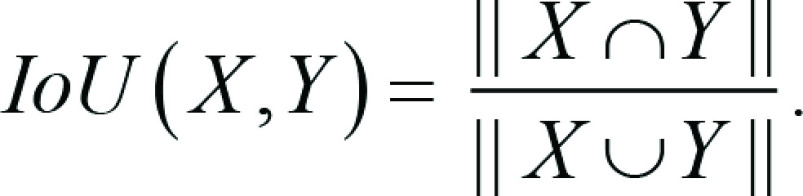


Dice score and IoU result in values between 0 and 1, where a value of 1 represents a perfect overlap between *X* and *Y*, and 0 represents no overlap.

To demonstrate the role of setting a baseline expectation when evaluating segmentation model performance, we developed a threshold-based approach to segment air inside the body for samples in the lung CT dataset as a proxy for lung segmentation to serve as a baseline model. Any sophisticated model for lung segmentation should outperform such a baseline.

We used any voxel with a Hounsfield unit value less than −400 as air. Then, we removed the segment of air outside the bodies of patients. This results in segmenting air within the body, which is used as an erroneous proxy for lung segmentation. We compared the performance of this simple model with the ground truth (lung contours). Any model with a performance less than such a baseline model should be considered irrelevant.

***Batch effects.—*** A batch effect occurs when data from several sources are aggregated to develop a larger dataset and the class distribution samples from these sources substantially vary. For example, suppose all malignant tumors in a cohort were imaged with MRI machine X and all benign tumors with MRI machine Y. In this case, the model may learn to differentiate malignant from benign tumors on the basis of MRI machine–attributed differences rather than intrinsic tumor characteristics. To investigate how batch effects impact model generalizability, we simulated a dataset with batch effect by extracting pneumonia samples from the dataset by Kermany et al (24) and the normal samples (ie, images with no findings) from the RSNA dataset. This dataset contains a batch effect, because the pneumonia samples come from children and the normal samples come from adults. Hereafter, we refer to this dataset as *Batch x-ray*. We trained a model (model I) on the Batch x-ray dataset. We then tested this model on an external dataset of normal chest radiographs from Kermany et al (24).

## Results

### Violation of Independence Assumption

Table 1 shows model performance when incorporating the described methodological pitfalls versus when these pitfalls are avoided. Oversampling was conducted before splitting data for model A and after splitting data for model B. The results showed a statistically significant gap between the performance of these two models. While model B—the correct approach—showed poor performance, model A seemed to offer promising results. Incorrect application of oversampling led to a statistically significant but superficial improvement in model performance (Wilcoxon rank sum test = −12.22, *P* < .001) for predicting local recurrence in head and neck cancer.

**Table 1:**
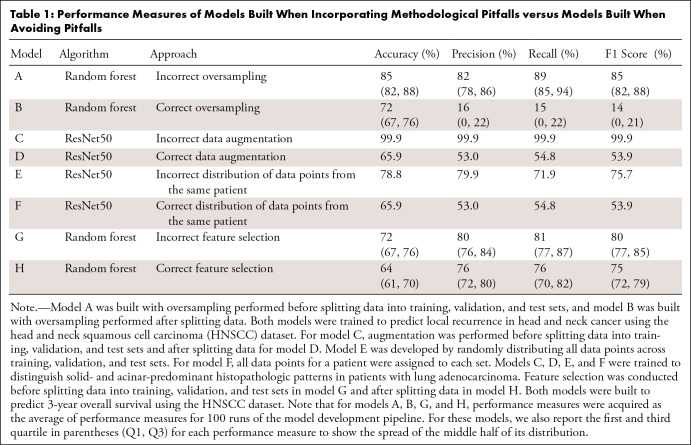
Performance Measures of Models Built When Incorporating Methodological Pitfalls versus Models Built When Avoiding Pitfalls

Models C, D, E, and F were built for distinguishing solid- and acinar-predominant histopathologic patterns in patients with lung adenocarcinoma. Due to the high computational cost and relatively larger dataset sizes for model development, we followed the common practice of reporting the results for one trained model; in contrast, for radiomics models A, B, G, and H, we repeated model building 100 times and reported average performance measures. Data augmentation was applied before and after splitting data for models C and D, respectively. Model C had seemingly higher performance than did model D.

The effect of breaking the independence assumption by distributing data points for a patient across training and test sets is demonstrated with models E and F. For model E, data points were randomly distributed across training, validation, and test sets. Therefore, data points for the same patient could appear in both training and test sets. For model F, the independence assumption was preserved by assigning data points for each patient to either the training, validation, or test set. Note that model F is the same as model D—that is, the correct approach for distinguishing solid- and acinar-predominant histopathologic patterns of the same patients with lung adenocarcinoma. Distributing data points of patients across training and test sets led to a seemingly higher performance of model E compared with model F.

Table 1 also shows how applying feature selection prior to splitting data into training, validation, and test sets could violate the independence assumption. Feature selection was conducted before data splitting for model G and after data splitting for model H. Applying feature selection before splitting data into training, validation, and test sets led to a significant superficial boost in model performance for predicting 3-year overall survival in head and neck cancer (Wilcoxon rank sum test = −5.87, *P* < .001).

### Model Evaluation with an Inappropriate Performance Indicator or Baseline for Comparison

A naive model that predicts all samples as nondistant metastasis would achieve an accuracy of 94% 
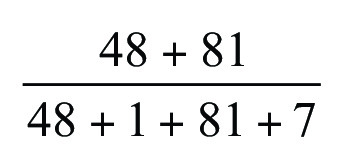
 for our HNSCC dataset. However, such a model has no medical use, as it achieves a recall of zero and fails to diagnose any distant metastasis. This example shows that using accuracy for highly imbalanced datasets might lead to developing models that cannot be deployed in a clinical setting.

**Figure 4:**
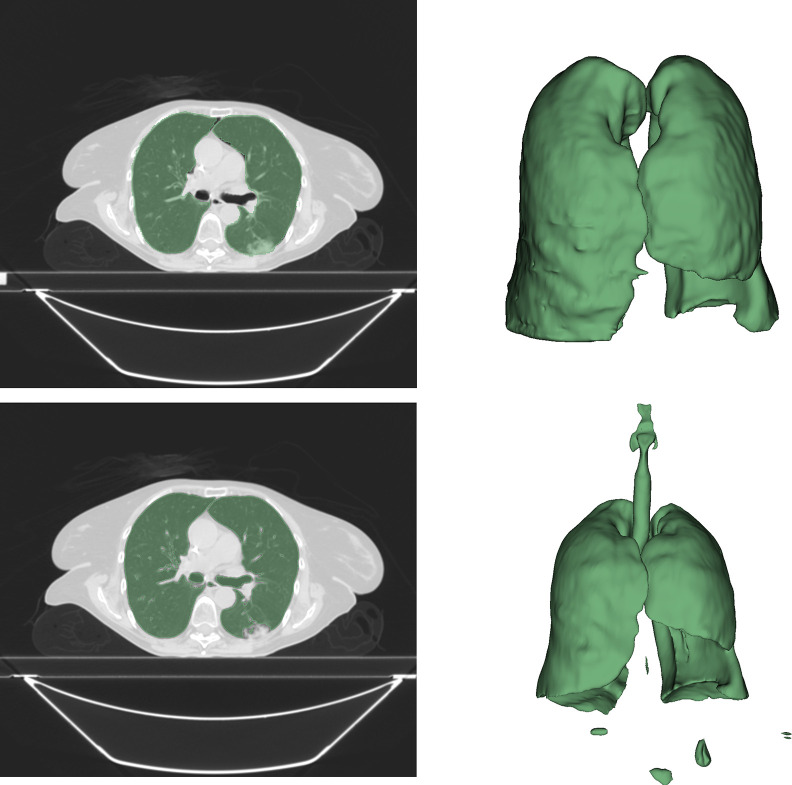
CT images of the lung with segmentation (shown in green). The top left image shows the ground truth mask overlaid on an axial section of a chest CT image manually contoured by a radiologist. The top right image illustrates the three-dimensional (3D) volume of the manual contour. The bottom left image illustrates a section of the predicted segmentation mask overlaid on its corresponding section of the CT image. The prediction has been made by a simple baseline model that detects air within the body. The bottom right image shows the 3D volume of the prediction made by the baseline model. The segmentation includes air in the body (including trachea and bowel gas), highlighting that for large volumes such as the lung, a high Dice score may not indicate high-quality segmentation.

Figure 4 illustrates the ground truth segmentation, as well as the predicted segmentation of a randomly chosen image from the lung CT dataset, where the prediction was made by a simple baseline model that detects air inside the body. While the predicted lung segmentation was not medically acceptable, for this example, the baseline model achieved a Dice score of 0.94 and an IoU of 0.88. Table 2 shows the minimum, mean, maximum, and SD of the baseline model performance measures when applied to samples in the lung CT dataset. The simple baseline model achieved a mean Dice score of 0.92 and a mean IoU of 0.86. Therefore, models with average performance measures lower than this baseline model should not be used.

**Table 2:**
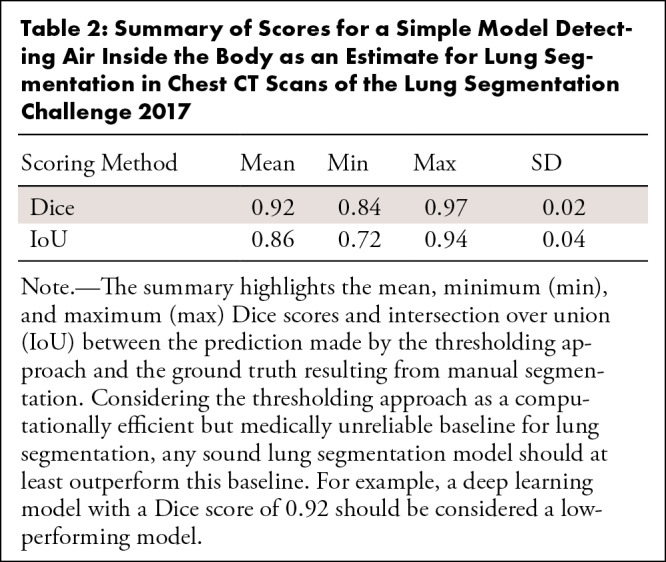
Summary of Scores for a Simple Model Detecting Air Inside the Body as an Estimate for Lung Segmentation in Chest CT Scans of the Lung Segmentation Challenge 2017

### Batch Effect

Our results also showed that batch effects could be a substantial barrier to model generalizability. We observed that model I, which was trained and tested on a dataset with batch effect (Batch x-ray dataset), achieved accuracy, precision, recall, and an F1 score of 99.7%, 97.9%, 99.5%, and 98.7%, respectively. However, when this model was applied to normal pediatric chest radiograph samples from the dataset by Kermany et al (24), only 3.86% of samples were correctly classified.

**Figure 5:**
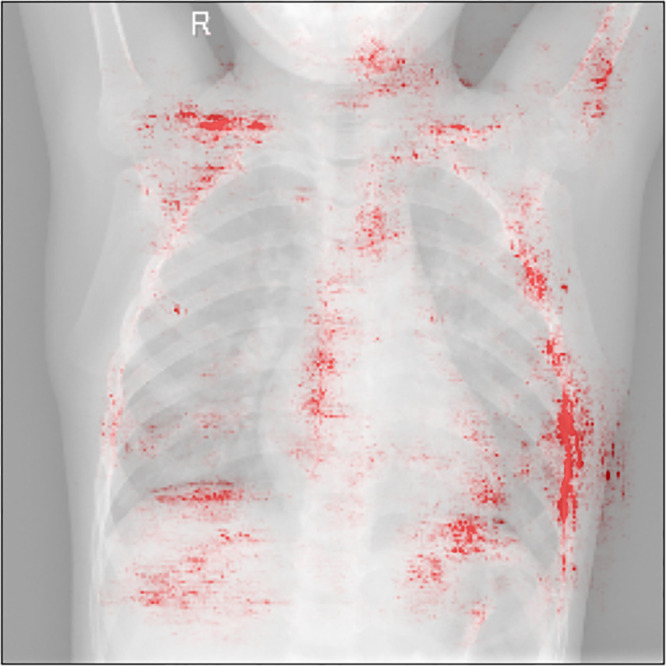
The attribution of each pixel of a radiograph in a healthy pediatric patient to model prediction is represented using integrated gradients. The radiograph pixels most attributed to model prediction are shown in red. The model, trained in the presence of a batch effect, incorrectly classifies this sample as pneumonia on the basis of anatomic structures and body position, which are substantially different between pediatric and adult patients.

The attribution of each image pixel to the model prediction for that image can be calculated using the integrated gradient method (27). Figure 5 overlays the attribution values for each pixel of a normal pediatric radiograph. The figure shows that the pneumonia prediction model trained using the Batch x-ray dataset focuses on anatomic structures and body position rather than image characteristics in the lung.

**Figure 6:**
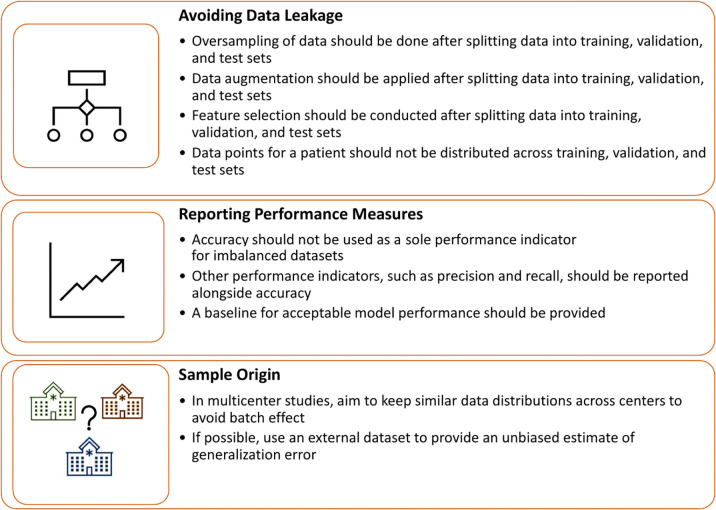
Methodological guidelines for developing generalizable machine learning and deep learning models.

Figure 6 presents a guideline to avoid the methodological errors discussed in this article.

## Discussion

With many recent studies highlighting the potential of ML and DL approaches for medical image analysis, the natural expectation is widespread use of these approaches in clinical settings. However, when applied prospectively, the lack of model generalizability is the main challenge. We investigated and highlighted some of the key methodological errors that lead to models that are not generalizable despite achieving deceptively promising results during internal evaluation. Such errors may be difficult to capture by readers, reviewers, or authors, especially if certain methodological details are not presented.

ML model performance is typically assessed using internal evaluation, where the available data are partitioned into training, validation, and test sets. To achieve an unbiased estimate of performance measures and generalization error for a model, data from the training and test sets must be independent. Any violation of the independence assumption should be avoided. When oversampling is conducted before randomly splitting data into training, validation, and test sets, the same copy of a data point could appear in training and test sets. Therefore, the training and test sets are no longer independent. Our results demonstrated that when oversampling was incorrectly applied to the HNSCC dataset, the model achieved superficially high performance; however, a correct approach led to poor results, as expected, due to the very small number of local recurrences in the dataset.

Similar superficial boosts were observed for incorrect application of data augmentation. When data augmentation is applied to an image, some of its characteristics change, but many will still be shared by the original and augmented images. If data augmentation is applied before data splitting, just as with oversampling, these highly correlated samples can be spread across the training, validation, and test sets, potentially leading to high performance on the internal test set and poor performance on external data.

Often, there are several data points associated with each patient. Extracting image patches is a common practice when analyzing histopathologic images or three-dimensional (3D) images (28). These patches might share characteristics irrelevant to the study goal. Distributing the different patches derived from a single patient between training, validation, and test sets could artificially boost model performance, but the resulting model will not be generalizable when applied to external data. Multiple data points for a single patient should be assigned to only the training, validation, or test set. For example, one should not assign a T2-weighted sequence of a patient to the training set and the corresponding T1-weighted sequence to the test set.

In radiomics, there are often several features representing the statistical characteristics, shape, and texture of a region of interest. Feature selection is an important step in developing ML models with high-dimensional features. If applied before splitting data, the information from all samples in the dataset is used to select a subset of features that work best for all samples. This partially exposes the test set, which will be selected in the next step, to the model and breaks the independence assumption. In this work, we observed that exposing the test samples to feature selection methods led to a superficial boost in performance measures. The selected features are discriminative for the given test set, leading to degraded performance on external data and a lack of generalizability.

Another key consideration in developing predictive models is selecting the appropriate performance indicators. For example, using accuracy for a diagnostic model for a rare condition is often misleading, as the model may disregard all cases of the rare condition in a dataset with high class imbalance and still achieve high accuracy. The cost associated with misclassification should also be considered. Consider a diagnostic test for a life-threatening condition. A method with high accuracy but low recall (sensitivity) cannot be used clinically. Similarly, high false-positive rates potentially leading to highly invasive procedures prohibit the deployment of predictive models in clinical settings. In such scenarios, precision should also be considered as a performance indicator to guide model development and evaluation.

Dice score and IoU are commonly used as performance measures for evaluating segmentation models. From a mathematical perspective, the Dice score is always larger than or equal to IoU (see Appendix S3 for a mathematical proof), which encourages reporting the Dice score. In our example in Figure 4, we observed that both metrics achieved a high value (IoU: 0.88 and Dice: 0.94), despite obvious flaws in lung segmentation. Therefore, we encourage visual inspection of segmentation model outcomes as a qualitative analysis. Additionally, pixel-level accuracy measurement should be avoided when evaluating small regions or volumes of interest.

During model development, it is desirable to collect and analyze imaging data from different sites. This is beneficial for increasing sample size and model generalizability because of the use of samples more representative of the target population. However, in addition to imaging techniques (29,30), the class distribution of samples substantially varies across sites. Thus, the aggregated dataset and resulting models could suffer from a batch effect. Of note, batch effect is sometimes disregarded. In a study of ML models used for COVID-19 diagnosis, Roberts et al (31) reported that some studies had used normal images from healthy pediatric patients, while the COVID-19 samples came from adults. Even for cases where training, validation, and test sets have the same distribution, the performance of a model should be regularly and systematically monitored. Due to the dynamic nature of data in real-world settings, the data distribution might change as time passes. This phenomenon—referred to as *distribution shift*, *domain shift*, or *domain drift*—might happen because of factors such as changes in imaging hardware, software, or protocols. In such cases, the performance of a model might degrade as time passes, demonstrating that training and evaluating ML and DL models is a nonstationary and iterative process.

Other ML and DL challenges beyond the described methodological errors, including data quality and availability, bias, and explainability, are outside the scope of this article. Recent literature covers potential AI misuse and provides suggestions and guidelines for how AI research can be used responsibly (31,32). Current guidelines also provide the framework for presenting ML and DL research to ensure the reproducibility of results (14). We recommend reviewing this literature for further knowledge, as the recommendations in this article are complementary to these works.

Developing ML and DL models for medical image analysis is challenging. Compared with natural images, medical images are often high dimensional due to their high resolutions (for example, in histopathologic analysis, whole-slide images) or their 3D nature (for example, in MR and CT images). Consequently, analyzing such data is challenging, specifically for domains where large-scale annotated datasets are unavailable. Among the challenges posed by high data dimensionality in medical imaging are the impracticality of whole-image analysis, the demanding nature of data annotation for medical images, and the increased possibility of overfitting. Whole-image analysis is impractical due to the limited amount of video random-access memory offered by graphics processing units. Therefore, patch-based approaches are used to analyze 3D and microscopic images, which in turn pose the challenge of combining the predictions made for these patches to achieve an image-level or patient-level outcome or prediction. This also complicates the data analysis pipeline and exposes the developed models to methodological pitfalls that might affect model generalizability. Pixel- or voxel-level annotation for medical images is also more tedious and time-consuming compared with that for natural images, as annotating a single 3D image often requires manual processing of hundreds of two-dimensional sections. Microscopic images also often have high resolutions, for example, 100 000 × 100 000 pixels, requiring greater effort to annotate each image. The increased possibility of model overfitting for high-dimensional data in the absence of large-scale datasets is also a major challenge affecting model generalizability. Due to these challenges, following best practices and avoiding methodological pitfalls is essential for developing generalizable models with the potential to be deployed in clinical settings. Developing methods tailored to medical images remains an active research area.

Medical image analysis is also interdisciplinary, requiring contributions from imaging, computational, and medical experts. Lack of expertise in one of these domains might lead to developing models that are not generalizable. For example, if medical expertise is available, it is unlikely that a comparison between pediatric samples and adult patients would be considered a valid experimental design given the substantial differences in anatomic and imaging components between these two groups. Furthermore, a model built to classify COVID-19 versus healthy lung using lung radiographs would immediately be recognized by a medical expert as requiring a more rigorous evaluation to ensure the model does not falsely detect other lung abnormalities as COVID-19. Collaboration and cooperation among various experts at each stage of medical image analysis are essential for the development of models that can ultimately be applied in a clinical setting.

This study had some limitations. While there are several DL architectures available that one could customize for medical image analysis, the models used in our study were limited to well-established architectures. This study could be further augmented by considering other model architectures and how susceptible they are to the violation of the independence assumption. Another important factor affecting the generalizability of ML and DL models, but not covered in this study, is the sample size used for model training and evaluation. Although there is general consensus on the positive impact of larger sample sizes and more varied datasets for training ML models, the literature could benefit from additional empirical analyses of the effect of sample size on model generalizability for future research. Another factor that has been reported to affect the performance of DL models (33,34), but not covered in this study, is image resolution. Using the National Institutes of Health ChestX-ray14 dataset, Sabottke and Spieler (33) studied the effect of image resolution on the performance of two widely used DL model architectures, ResNet34 (35) and DenseNet121 (33,36). Investigating different image resolutions ranging from 32 × 32 to 600 × 600 pixels, they showed that the optimal selection of image resolution is task dependent and essential for increasing model performance in several classification tasks. Last, in this investigation, we mainly focused on the methodological errors resulting from violation of the independence assumption. However, another critical requirement for developing generalizable models is that the datasets used for model training and evaluation should represent the distribution of the data in a real-world setting (ie, data at the deployment phase), which may not always be possible. For example, samples from minority classes might be absent or poorly represented in the test set. In such a case, a model that works well on prevalent classes but poorly on minority classes still achieves high performance measures when evaluated using such a test set. Techniques such as stratified data splitting could be used to ensure similar representations for only known classes or covariates, not unidentified covariates, across training, validation, and test sets. The problem is more pronounced when using small sample sizes, highlighting the need for developing large and diverse datasets for medical image analyses. One must ensure that best practices in ML model development are followed despite restricted access to patient data and source code.

In conclusion, we studied several methodological pitfalls in developing ML and DL models. These pitfalls are undetected during the internal evaluation of models, leading to overoptimistic estimations of model performance and consequent lack of generalizability. Awareness of these pitfalls and consideration of the suggested guidelines for avoiding them are important for developing generalizable ML and DL health care models.
